# Pilomatrixoma of Earlobe

**DOI:** 10.4103/0974-2077.53099

**Published:** 2009

**Authors:** Mohamed Jallouli, Houssem Yengui, Abdelmajid Khabir, Riadh Mhiri

**Affiliations:** *Department of Pediatric Surgery, Hedi Chaker Hospital, Sfax, Tunisia*; 1 *Department of Pathology, Habib Bourguiba Hospital, Sfax, Tunisia*

**Keywords:** Calcification, earlobes, pilomatrixoma

## Abstract

Pilomatrixomas are uncommon in children and are frequently misdiagnosed preoperatively. We report a two-year-old female patient with an unusual localization in the earlobe. The lesion was treated by simple enucleation and in two years of follow-up there has been no evidence of recurrence. The case is being reported in view of its rarity and unusual location.

## INTRODUCTION

Pilomatrixomas are uncommon tumors which are frequently misdiagnosed preoperatively. The tumor usually appears in the first two decades of life and is commonly located in the head and neck region.[1,2] Few cases of pilomatrixoma in the auricular area and earlobe have been reported.[3] We report a case of pilomatrixoma of earlobe.

## CASE REPORT

A two-year-old girl presented with a firm swelling in the earlobe of eight months duration. There was history of trauma to the area. Initially, the swelling was red and painful; hence, patient was thought to have an abscess and treated with systemic antibiotics but in vain. Lesion gradually increased in size and there was spontaneous resolution of redness.

**Figure 1:**
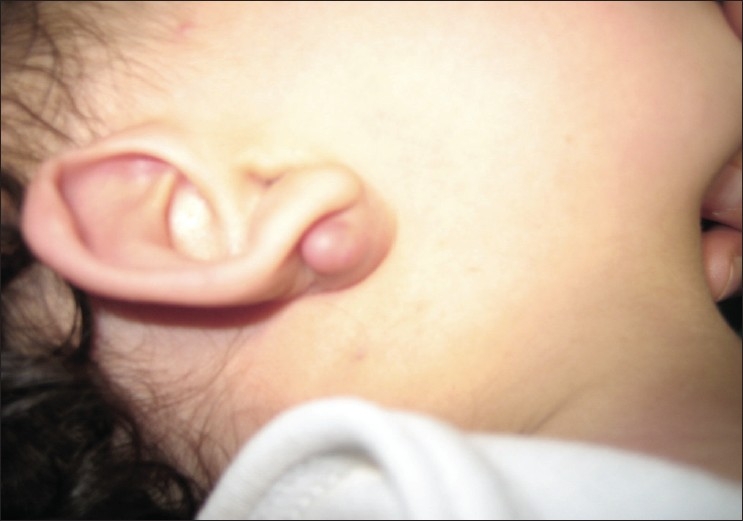
Auricular mass (Note the absence of inflammatory sign)

Examination of the left earlobe revealed a non-tender, firm, well-circumscribed, freely mobile nodule located below the right ear nodule [Figure 1]. The overlying skin was normal in appearance. The surgical procedure consisted of enucleation of the lesion. At the time of surgery, the tumor was easily shelled out of the surrounding tissues. The surgical specimen revealed a hard irregular mass measuring 1.5 × 1 × 0.5 cm. The histopathologic exam confirmed the diagnosis of pilomatrixoma [Figure 2a and 2b]. Patient has been followed up for two years with no recurrences and satisfactory cosmetic result [Figure 3].

**Figure 2a:**
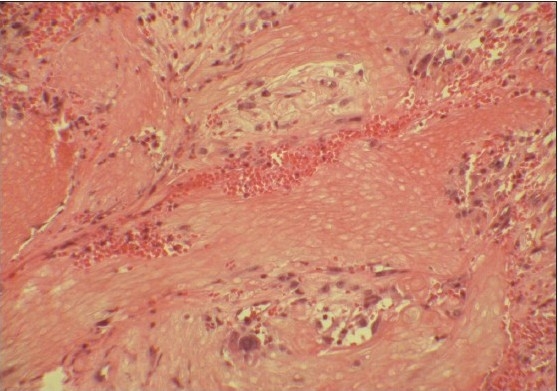
Skin biopsy reveals masses of mummified cells with a stromal inflammatory reaction with giant cells (H and E, ×100)

**Figure 2b:**
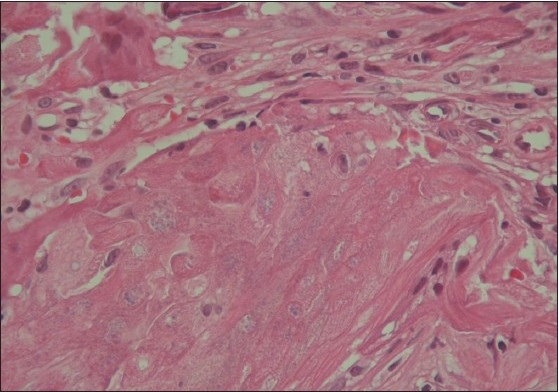
Higher magnification of the mummified cells (H and E, ×400)

**Figure 3:**
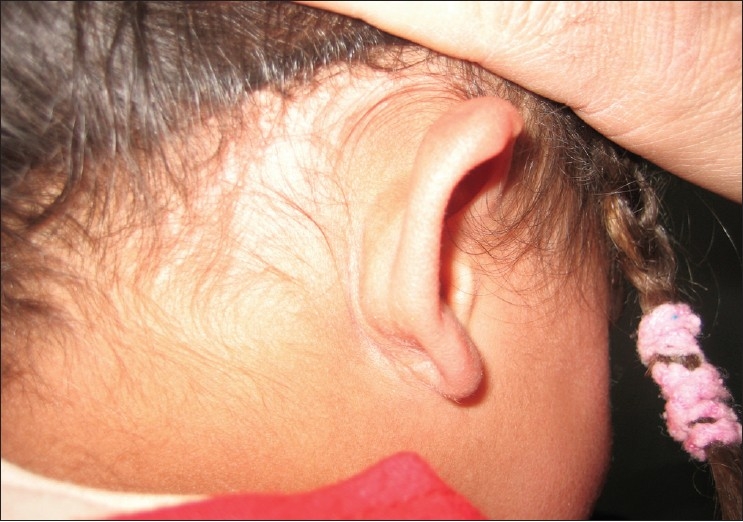
Post-treatment photographs showed a good aesthetic result

## DISCUSSION

Pilomatrixoma is a relatively rare, benign skin tumor arising from the hair follicle. Pilomatrixoma occurs at any age, but is most common in children under the age of ten.[1] Women[2] and White race[4] are more prone for pilomatrixoma.

Although pilomatrixoma is common in the head and neck region,[1,2] it can also be found in the scalp, eyelids, arms and preauricular area.[1–3] It typically presents as a slow-growing, superficial, firm, asymptomatic, freely mobile mass of the dermis. It is usually solitary, but multiple lesions have been reported.[5,6] Inflammation or hemorrhage into the neoplasm may give rise to sudden increase in size.

Pilomatrixoma develops from an abnormal formation of cells that are similar to hair cells, which become hardened or calcified. The etiology is not completely understood, though the role of activating mutation in the b-catenin gene mapped to Chromosome 3p22-21.3 has been reported.[7,8] Pilomatrixoma may rarely be familial and such familial cases may be also observed in association with disorders such as Gardner syndrome, Steinert disease, sarcoidosis, myotonic dystrophy, Turner syndrome, xeroderma pigmentosum.[1,2,8]

Biopsy is the gold standard for diagnosis of this condition;[7] fine needle aspiration cytology is not adequate and therefore not recommended for diagnostic purpose.[9]

The treatment of choice is local excision.[1,2] Recurrence after complete surgery is rare, as in our case. Malignant transformation has not been reported in children.[10,11]

## CONCLUSION

Physicians, otolaryngology's, dermatologists and surgeons should be familiar with this entity of pilomatrixoma and consider it in the differential diagnosis of superficial or calcified subcutaneous masses in the auricular region.
